# Molecular Characterization of *Streptococcus agalactiae* Isolated from Bovine Mastitis in Eastern China

**DOI:** 10.1371/journal.pone.0067755

**Published:** 2013-07-10

**Authors:** Yongchun Yang, Yinglong Liu, Yunlei Ding, Li Yi, Zhe Ma, Hongjie Fan, Chengping Lu

**Affiliations:** College of Veterinary Medicine, Nanjing Agriculture University, Nanjing, China; Beijing Institute of Microbiology and Epidemiology, China

## Abstract

One hundred and two *Streptococcus agalactiae* (group B streptococcus [GBS]) isolates were collected from dairy cattle with subclinical mastitis in Eastern China during 2011. Clonal groups were established by multilocus sequence typing (MLST) and pulsed-field gel electrophoresis (PFGE), respectively. Capsular polysaccharides (CPS), pilus and alpha-like-protein (Alp) family genes were also characterized by molecular techniques. MLST analysis revealed that these isolates were limited to three clonal groups and were clustered in six different lineages, i.e. ST (sequence type) 103, ST568, ST67, ST301, ST313 and ST570, of which ST568 and ST570 were new genotypes. PFGE analysis revealed this isolates were clustered in 27 PFGE types, of which, types 7, 8, 14, 15, 16, 18, 23 and 25 were the eight major types, comprising close to 70% (71/102) of all the isolates. The most prevalent sequence types were ST103 (58% isolates) and ST568 (31% isolates), comprising capsular genotype Ia isolates without any of the detected Alp genes, suggesting the appearance of novel genomic backgrounds of prevalent strains of bovine *S. agalactiae*. All the strains possessed the pilus island 2b (PI-2b) gene and the prevalent capsular genotypes were types Ia (89% isolates) and II (11% isolates), the conserved pilus type providing suitable data for the development of vaccines against mastitis caused by *S. agalactiae*.

## Introduction

*Streptococcus agalactiae*, also referred to as group B streptococcus (GBS), is one of the leading causes of bovine mastitis, which has economically important implications for the dairy cattle industry throughout the world [1]. *S. agalactiae* is also an important human pathogen that can induce invasive infections in neonates, the elderly and pregnant women [2,3,4]. There is indirect evidence that *S. agalactiae* is transmitted between cattle and humans [5]. Clearly, control and prevention of *S. agalactiae* mastitis will improve the quantity and quality of milk production and have important significance for animal welfare and public health. As we know, studies of the molecular epidemiology of field *S. agalactiae* strains is vitally important in implementing efficient management practices in dairy farms. However, no information regarding the molecular characterization of *S. agalactiae* strains occurring in farms in China has previously been documented.

Multilocus sequence typing (MLST) and pulsed-field gel electrophoresis (PFGE) typing are two genotype methods used to characterize and distinguish specific clones among GBS isolates [6]. MLST is an unambiguous sequence-based and reliable typing tool, allowing comparison of the gene distribution of different isolates collected from all geographic areas and further investigation of the population structure [6,7]. Five hundred and eighty-three *S. agalactiae* sequence types (STs) have been identified and made available on the MLST website as of May 2012 (http://pubmlst.org/sagalactiae/), although information about bovine strains is still limited.

Capsular serotyping is a classical method used for *S. agalactiae* in epidemiological studies. To date, ten serotypes, based on the *S. agalactiae* capsular polysaccharides (CPS), have been identiﬁed, including Ia, Ib, II-VIII and a new serotype IX [8]. Capsular genotyping is considered more suitable for epidemiological investigation because the serotypes can be identified with or without the CPS expression.

The alpha-like protein (Alp) family play an important role in *S. agalactiae* pathogenesis and are also vaccine candidates [9]. Six members of the Alp family have been extensively studied, including Alpha-C, Rib, Alp1 (Epsilon), Alp2, Alp3 and Alp4 [10], they are encoded by genes *bca*, *alp1* (*Epsilon*), *alp2/3*, *Rib*, and *alp4*, respectively.

Three types of pili of *S. agalactiae* have been identified and designated as pilus island 1 (PI-1), PI-2a and PI-2b, of which PI-2a and PI-2b are encoded by genes located in two distinct loci in the same region of the genome, while the PI-1 gene is located in a separate region [11].

The objectives of this study were to elucidate the relationships between bovine *S. agalactiae* strains by molecular characterization based on capsular genotyping, pilus and Alp gene profiling and MLST and PFGE analyses.

## Results

### Identification of *S. agalactiae* in dairy cows

Milk samples were collected from a total of 619 cows with subclinical mastitis from 33 dairy farms located in six provinces (Jiangsu, Anhui, Shandong, Zhegjiang, Jiangxi and Fujjian) and one municipality (Shanghai). *S. agalactiae* was detected in milk samples from 102 cows from 21 farms located at five provinces (except Fujian province) and one municipality. In total, 102 bovine isolates (one isolate per cow) of *S. agalactiae* were selected and analyzed (a complete list of all isolates is provided in Table S1).

### Capsular genotype and alpha-like protein

All 102 bovine *S. agalactiae* isolates belonged to capsular genotype Ia and II (Table 1) according to the multiplex PCR assay. Overall, type Ia was the most prevalent accounting for approximately 89% (91/102) of isolates. Type II was represented by 11% (11/102) of isolates. A large proportion (93%; n = 95/102) of the bovine *S. agalactiae* isolates was non-typeable (NT) to any of the detected Alp genes according to the multiple PCR assay (Table 1). Only 5% (5/102) of Alp1 gene-positive strains and 2% (2/102) Alp4 gene-positive strains were characterized. The alpha-like protein genes associated with the capsular genotype were also identified (Table 1): *alp1* and *alp4* associated with genotype II, NT associated with genotype II and Ia (χ^2^=36.595, *P* < 0.001).

**Table 1:** CCs, STs, capsular genotypes, pilus types, alpha-like protein genes and PFGE types of the 102 bovine *S. agalactiae* strains.

CCs^a^ and ST^b^	Allelic profile^c^							Capsular genotype (No. of isolates)	Frequency (%)	PI^d^	Alp^e^ gene (No. of isolates)	PFGE type (No. of isolates)
CC67									10			
67	13	1	1	13	1	1	5	Ia (1)	1	2b	NT^f^ (1)	3(1)
								II (4)	4	2b	NT (4)	3(1), 4(1), 8(1), 13(1)
301	13	1	1	13	1	28	5	II (4)	4	2b	*alp*1 (4)	1 (1), 11 (1), 12 (1), 22 (1)
313	13	1	1	2	1	28	5	II (1)	1	2b	*alp*1 (1)	11 (1)
CC64									2			
570	16	1	1	2	1	1	5	II (2)	2	2b	*alp*4 (2)	9(2)
CC103									88			
103	16	1	6	2	9	9	2	Ia (58)	57	2b	NT (58)	6 (1), 7 (25), 8 (5), 10 (3), 14 (8),) 15 (7), 17 (1), 18 (5), 19 (3)
568	16	1	6	2	51	9	2	Ia (32)	31	2b	NT (32)	2 (1), 5 (1), 12 (2), 16 (6), 20 (1), 21 (1), 23 (5), 24 (2), 25 (9), 26 (3), 27 (1)

^a^ CC, clonal complex. ^b^ ST, sequence type. ^c^ The allelic proﬁles are presented in the following order: *adhP, pheS, atr, glnA, sdhA, glcK, and tkt*. ^d^ PI, pilus island. Minus in this column indicates the absence of a PI-1 gene. ^e^ Alp, alpha-like protein. ^f^ NT, non-typeable.

### Pilus type

Each strain was detected in the three PCR assays for pilus typing. PI-1 and PI-2a genes were absent in all of the investigated bovine strains although the corresponding genes were detected in the positive controls. All 102 bovine *S. agalactiae* strains carried the PI-2b gene alone (Table 1).

### MLST sequence types and PFGE analysis

**Figure 1:**
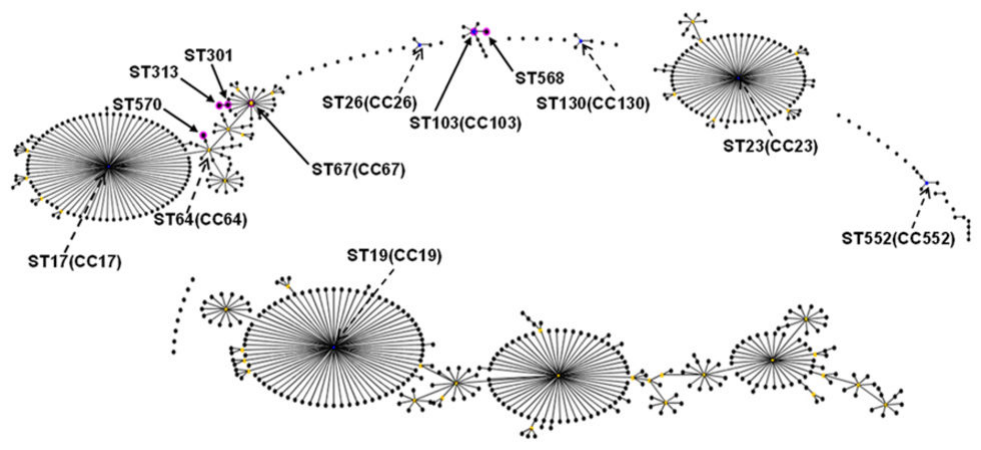
eBURST diagram of the *Streptococcus agalactiae* population. Each sequence type (ST) is represented as a dot. The dots positioned centrally in the cluster are primary founders (blue) or subgroup founders (yellow). Pink circles indicate STs detected from the strains in this study and are marked by solid line arrows. The 102 bovine isolates were clustered within sequence types ST103, ST568, ST67, ST301, ST313 and ST570 and grouped within three clonal complexes (CCs): CC103, CC67 and CC64. Both ST67 and ST64 were subgroups of CC17. ST568 was a single-locus variant (SLV) for ST103. ST301 and ST313 were SLVs and DLVs (two-locus variants), respectively of ST67. ST570 was a DLV of both ST64 and ST67, derived from ST64. The predicted founders that were not obtained in this study are marked by dashed arrows. For clarity, ST labels have been removed.

All 102 isolates were characterized using MLST; one novel allele (*sdh*A allele 51, GenBank accession number is KF006268) and two new STs (ST 568 and ST570) were identified. ST568 is a single-locus variant (SLV, in which one allele differs from the ST) of ST103, ST570 is a double-locus variant (DLV, in which two alleles differ from the ST) of ST64 and ST67 according to eBURST analysis. ST568 was assigned based on the presence of a novel allele (*sdhA* allele 51) and ST570 was assigned based on new combinations of allele profiles. In general, all 102 bovine isolates were clustered in six STs: ST103, ST568, ST67, ST301, ST313 and ST570, and were grouped in three clonal complexes (CC): CC67, CC64 and CC103 by eBURST analysis (Figure 1). Figure 1 also shows the close correlation between CC67 and CC64, which are both subgroups of CC17. This was further supported by the relationship between ST570 and ST67 (ST570 was a DLV of ST67) as described previously. However, CC103 remained as a distinct group.

**Figure 2:**
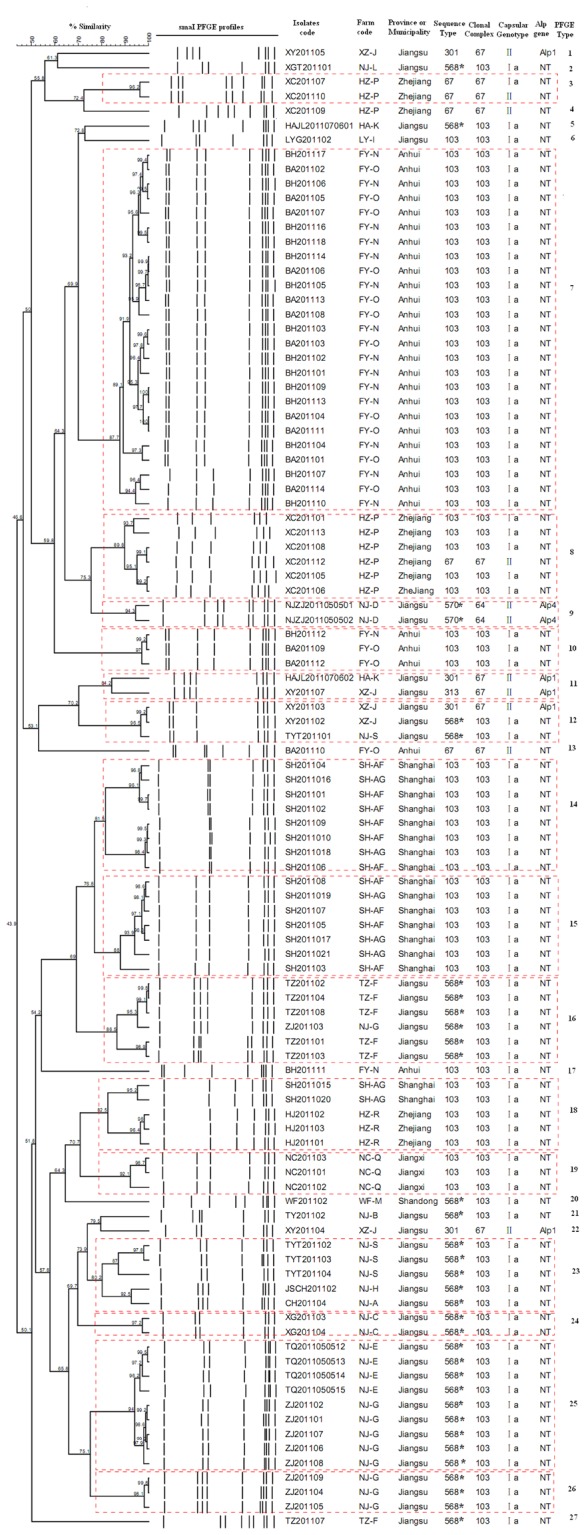
Dendrogram of PFGE profiles of 102 bovine *Streptococcus agalactiae* The Dendrogram was constructed based on BioNumerics analysis of the *S. agalactiae* PFGE patterns and cluster analysis using the Dice coefﬁcient and UPGMA of the digitalized PFGE patterns for the *S. agalactiae* strains. Clustering settings of 0.00% optimization and 1.5% band position tolerance were used. 568 * and 570* were identified as novel sequence types in this study.

The isolates were grouped in 27 PFGE types in this study, of which 16 common (represented by dashed rectangles) and 11 unique types were identified, with a similarity between 43.9% and 100% (Figure 2). Eight major PFGE types (7, 8, 14, 15, 16, 18, 23 and 25 shown in Figure 2) comprised close to 70% (71/102) of all the isolates. A total of eight more minor types, comprising only two or three isolates, corresponded to almost 20% (20/102) of all the isolates.

Table 1 shows the 11 capsular genotype II isolates that were distributed among four STs (ST67, ST301, ST313, ST570) and eight PFGE types. The 91 type Ia isolates belonged to three STs (ST67, ST103, ST568) and 21 different PFGE types. ST103 and novel sequence type ST568, both belonging to CC 103 (Figure 1), were the predominant sequence types. These types comprised genotype Ia isolates associated with NT *Alp* genes and represented 57% (58/102) and 31% (32/102) of the isolates, respectively (Table 1). These isolates were clustered into 20 types according to the PFGE analysis. ST67, ST313 (DLV of ST67) and ST301 (SLV of ST67), were grouped in CC67 (Figure 1). ST67 represented 5% (5/102) of the isolates assigned to capsular genotype II (4 isolates) and Ia (1 isolate) associated with NT *Alp* genes, and were further clustered in four PFGE types. ST313 and ST301 comprised a total of 5% (5/102) of the isolates, which were assigned to capsular genotype II carrying *Alp1* genes and belonged to five PFGE types. ST570, belonging to CC64, represented only two isolates of serotype II carrying the *Alp4* gene and clustered in PFGE type 9 (Table 1).

The isolates clustered in the same ST were clearly divided into several distinct PFGE types. For example, ST103 and 568, were divided into nine and eleven different PFGE types, respectively (Table 1 Figure 2). It was also were observed that the isolates belonging to different capsular genotypes were clustered in the same ST or PFGE type, while the same capsular genotypes were divided into different ST or PFGE types (Table 1 Figure 2).

### Distribution of *S. agalactiae* between and within farms

**Figure 3:**
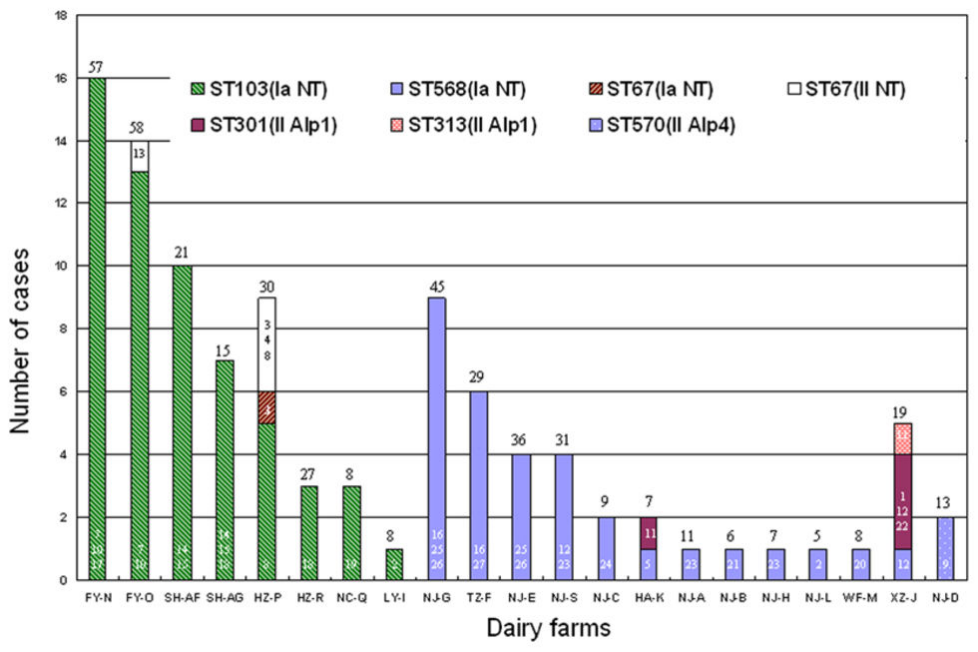
Typing of 102 *Streptococcus agalactiae* isolates present in 21 commercial dairy farms in Eastern China. The percentage of *Streptococcus agalactiae* positive cases is shown above each bar. ST indicates the MLST sequence type; the corresponding capsular genotype/alpha-like protein type is shown in parentheses. NT, non-typeable. The PFGE types are represented by the Arabic numerals in each bar.

The STs, molecular genotypes, Alp genes of *S. agalactiae* among the 21 *S. agalactiae* positive farms are presented in Figure 3. Seven different genotypes were found when combining these three types. The predominant type, ST568, a novel ST which is a SLV of ST103, combined with Ia and NT and was found exclusively among isolates from farms located in northern provinces (Jiangsu and Shandong) only. ST103, the predicted founder of ST568, combined with Ia and NT, and with few exceptions, was found exclusively among isolates in the farms located in the southern provinces (including Shanghai, Anhui and Zhejiang).

On the basis of the combination of genotypes described, the isolates were characterized into 31 types when combined with the PFGE genotypes (Figure 3). Comparison of *S. agalactiae* isolates between herds revealed similar genotypes (10 types) distributed in different herds, and herd specific types (21 types) were also observed (Figure 3). Comparison of *S. agalactiae* isolates from the same herd showed *S. agalactiae* isolates displaying either different types (in 12 herds) or the same type (in 9 herds) within a same herd. Clustering of multiple isolates of the same type within an individual herd was also observed on several farms.

## Discussion

In the present study, 21 of 33 dairy farms screened positive for *S. agalactiae*, although control measures were managed in these farms. It was reported that the herd level prevalence of *S. agalactiae* increased steadily from 2000 to 2008 in Denmark, even with systematic control measures in place, which had previously been effective in preventing *S. agalactiae* mastitis [12]. The increasing prevalence may be due to an increase infection rate that exceeds the capacity for eradication. This demonstrates that *S. agalactiae* remains a significant cause of mastitis in cattle herds, and more effective management is required to control *S. agalactiae* mastitis.

The CPS of *S. agalactiae* are one of the most important virulence factors and form the main components of multivalent vaccines [13]. Capsular genotyping by multiplex PCR revealed that Ia was the most predominant capsular genotype in Eastern China. Similar findings have been reported in Germany where serotype Ia was shown to be prevalent in 19 of 79 bovine *S. agalactiae* isolates [14]. However, serotype III and serotypes V and IV were found to be the most prevalent in Quebec (Canada) and Norway, respectively [15,16]. The diversity in serotype distribution of mastitis *S. agalactiae* might be the result of divergent geographical regions, times, management practices and breeds of cow. In the present study, all the bovine *S. agalactiae* isolates from 21 farms of five provinces and one municipality in Eastern China belonged to serotype Ia and II.

The major surface-localized protein antigens of *S. agalactiae* belong to the alpha-like protein (Alp) family of surface proteins. It is reported that *S. agalactiae* strains usually carry at least one of the alpha-like proteins, of which proteins Epsilon (Alp1), Alp2, Alp3, Alp4, Alpha-C and Rib have been extensively studied [17], although several bovine and human strains have been reported to be negative for any of the six proteins [16,17,18]. In this study, the multiple PCR assay for detecting Alp genes showed that 92% of the tested bovine isolates were non-typeable, possibly due to mismatch of the primers for genes encoding alpha-like proteins in these isolates. Many studies have previously shown the associations of Alp genes and serotypes; for example, the Alpha-C protein gene with serotypes Ia and II [17,19], the Epsilon/alp1 gene with serotype Ia [19] and the Rib gene with serotype II [10]. However, in this study, different combinations of Alp genes and capsular genotypes were found; the Epsilon/alp1and alp4 genes were signiﬁcantly associated with type II, and NT Alp genes with type Ia and II. The novel combinations of Alp genes and serotypes are suggested by the emergence of new *S. agalactiae* clones [6].

*S. agalactiae* strains carry at least one of the three pilus islands. Margarit et al. [20] demonstrated that all 289 human strains they investigated carried either PI-2a (73% strains) or PI-2b (27% strains), while PI-1 was missing in 28% of the strains. Following analysis of 238 isolates, Sørensen and colleagues [21] found that all the isolates carried PI-2, but PI-1 was missing in 54% of the bovine isolates and in 24% of the human isolates; therefore, it was concluded that PI-1 genes do not exist in several lineages. In this study, all of the 102 bovine *S. agalactiae* strains from Eastern China carried only PI-2b, while PI-1 was absent from all of the strains. Since previous studies have shown that pilus-based vaccines can be effective in preventing infections caused by homologous challenge of *S. agalactiae* [20], PI-2b proteins may be considered as potential vaccine candidates for formulation of subunit vaccines against bovine *S. agalactiae* mastitis.

Two novel sequence types, ST568 and ST570, were found in this study, which was expected because the information in the MLST database about the *S. agalactiae* isolates prevailing in China is severely lacking. Thus, the submission of our data to this database enriches the available data on bovine *S. agalactiae* isolates. The novel sequence type ST568 and its predicted founder ST103 were the predominant STs found in this study. ST103 is occasionally obtained from dairy cows according to previous reports [22]. However, a recent study shows ST103 to be a predominant ST in bovine strains from Denmark [23] and here it is shown to be prevalent in Eastern China, which contributes to a better understanding of the global epidemiology of mastitis *S. agalactiae* isolates. The new sequence type ST570, which is a SLV of ST 64, represented only 2% of the isolates in this study. Conversely, its predicted founder ST64 has been reported as a common ST among bovine *S. agalactiae* strains [21]. ST67, ST301 and ST313 were grouped in CC67, representing only a total of 12% of the isolates in this study. However, ST67 has previously been considered to be the most common ST among bovine isolates [2]. The occurrence of unique and identical STs identified between Eastern China and other countries shows that the types of ST and predominant STs differ in bovine isolates from divergent geographical regions. However, limited clonal groups appear in different region and countries according to MLST analysis. In present study, CC67 and CC64 showed a high degree clonal of relatedness, while CC103 remained as a distinct group, suggesting the *S. agalactiae* strains from bovine milk in Eastern China comprise two genetically distinct populations.

In this study, identical capsular genotypes belonged to different clonal groups and identical STs, PFGE types shared by various capsular genotypes were observed. This switching may be the result of horizontal transfer of capsular genes, which is likely to be driven by the host immune response and supported by the increased ﬁtness acquired by isolates showing specific phenotype-genotype combinations [6,24].

Similar sequence types prevalent on several different farms were observed in this study. The novel ST568 is prevalent in the north, while ST103, the predicted founder of ST568, is predominant in the south of this region, thus indicating that the prevailing *S. agalactiae* on these related farms are from the same strain, and might be transmitted from farms in the south to those in the north. It can be speculated that this occurs as a result of the commercial movement of infected animals between farms. Further analysis using PFGE demonstrated that the isolates grouped in the same ST were divided into several different PFGE types. The combination of genotypes presented several phenomena: farm specific in several farms, homology of the isolates among several farms, heterogeneity in isolates within several individual herds and multiple cows infected by a single strain on the same farms. *S. agalactiae* is a well-known contagious mastitis pathogen, with transmission occurring between cows within herds [1]. This mode of transmission is thought to explain how a single strain prevails in the same herd in the present study and in previous reports [25]. Different PFGE types were found in isolates clustered in the same STs, which may be accounted for by different management practices between farms. Homology of isolates in different farms and heterogeneity in isolates within individual herds are reported for the first time in this study. As previously speculated, the homology of isolates in different farms might also be due to commercial movement of infected animals between farms; however, it is not known why and how several different genotypes of *S. agalactiae* strains were obtained from the same herd, because few molecular epidemiological studies of sources of infection or transmission routes have been conducted in cattle. This heterogeneity might suggest the emergence of several strains originating from several sources of infection, with infected humans as the suspected infection source [26].

In conclusion, the results of the present study demonstrate that the distribution of STs, capsular genotypes, and pili genes among the dairy cattle in Eastern China were similar to previously reports of bovine *S. agalactiae* strains; however some geographic characteristics were revealed by the emergence of unique Alp profiles and prevalent novel STs. ST103 to be a predominant ST in bovine mastitis from Eastern China, which contributes to a better understanding of the global epidemiology of mastitis *S. agalactiae* isolates. The conserved pilus type of *S. agalactiae* isolates provides positive information for the development of vaccines against *S. agalactiae*. Further investigation is necessary to establish the epidemiology of *S. agalactiae*, and to determine how *S. agalactiae* can be best controlled and prevented.

## Materials and Methods

### Ethics

Milk samples were obtained with consent from animals with subclinical mastitis under the ethical approval granted by College of Veterinary Medicine, the Nanjing Agricultural University Veterinary College. The protocol was permitted by the owners of the dairy farms under investigation. All efforts were made to minimize animal suffering.

### *S. agalactiae* reference strains

Four reference strains of *S. agalactiae*: ATCC 13813 (serotype II), ATCC 12403 (Serotype III), ATCC BAA-611 (Serotype V) and A909 (Serotype Ia), used in this study were obtained from the American Type Culture Collection (ATCC) as controls.

### Identification of *S. agalactiae* from milk samples

A total of 619 cows from 33 large-scale diary farms located in Eastern China were selected to participate in this study according to the willing of the farmers (information about the farms were provided in Table S2). Holsteins dairy cows were preferably kept on the 33 dairy farms with a mean yield at 305 d of 7,539 kg of milk. For each farm, milk somatic cell counts (SCC) of each cow were routinely carried out twice per month by electronic counting (Fossomatic 5000^TM^, Foss Electric, Hillerød, Denmark). Subclinical mastitis is suspected when SCC ≥300,000 cells/ml, with decreased milk production and no inflammation of the udder. Milk samples were aseptically collected from dairy cattle with subclinical mastitis in these farms from January to December in 2011. In brief, each udder of the subclinical mastitis cow was washed and dried with a clean face towel. Each teat was disinfected with swabs soaked in 70% ethyl alcohol. The first few streams were discarded before milk samples were collected in 10-ml sterile plastic tubes and numbered. Each teat was disinfected with 70% ethyl alcohol after collection. The samples were taken to the laboratory as soon as possible, or stored at -20°C for later analysis.

For isolation of *S. agalactiae*, 20 µl of milk was streaked on 5% sheep blood agar plates and incubated at 37°C for 24 h. *S. agalactiae* were identified by conventional methods [27] and were further confirmed by PCR with 16S DNA species-speciﬁc primers as described by Martinez et al. [28]. A single isolate from each sampled cow was selected and stored at -70°C for further experimental use.

### Bacterial culture and DNA extraction

The isolates, including the reference strains, were cultured in 3 ml of Tryptic soya broth (TSB, MO BIO, Laboratories. Inc.) overnight at 37°C. The bacterial culture was centrifuged (14,000 ×*g* for 5 min at room temperature) and the pellets were harvested and resuspended in 200 µl TE buffer (10 mM Tris-HCl, pH 8.0; 1 mM EDTA, pH 8.0) supplemented with 20 mg/ml (final concentration) of lysozyme and incubated at 37°C for 30 min. Genomic DNA was extracted using the Bacterial DNA extraction kit following manufacturer’s specifications for Gram-positive bacteria (Omega Bio-Tek, USA). The extracted DNA was used as the template for PCR.

### Capsular genotyping

The capsular genotype Ia, Ib, II–IX of *S. agalactiae* was identified by a multiplex PCR assay as previously described [29]. The PCR system (total volume, 25 µl) contained 50 ng DNA template, 1× PCR buffer; 2 mmol/1 MgCl_2_, 200 µmol/l dNTPs (dATP, dTTP dCTP, and dGTP), 400 nmol/l primers cpsI-Ia-6-7-F and cpsI-7-9-F, 250 nmol/l of each other primer and 0.3 U of HotMaster Taq DNA Polymerase (Tiangen, China). The PCR amplification conditions were as follows: preheating at 95°C for 5 min, followed by 15 cycles of 95°C for 1 min, 54°C for 1 min, and 72°C for 2 min and then by an additional 25 cycles of 95°C for 1 min, 56°C for 1 min, and 72°C for 2 min with a final cycle of 72°C for 10 min. Serotypes of strains were identified by analyzing the unique banding pattern following agarose gel (1.5% wt./vol.) electrophoresis.

### Alpha-like protein (Alp) family

The alpha-like protein genes *bca*, *alp1* (*Epsilon*), *alp2/3*, *Rib*, and *alp4* in the strains were detected by a simple multiplex PCR assay described by Creti, et al. [17]. In brief, the PCR system (total volume, 25 µl) contained the following: 50 ng DNA template, 1× PCR buffer; 2 mmol/l MgCl_2_, 200 µmol/l dNTPs, 400 nmol/l of each of the five pairs of primers; 0.3 U of HotMaster Taq DNA Polymerase (Tiangen, China). The amplification conditions were as follows: preheating at 96°C for 3 min, followed by 30 cycles of 95°C for 1 min, 58°C for 45 s, and 72°C for 45 s, with a ﬁnal cycle of 72°C for 10 min. Amplification of the alpha-like protein genes was evaluated by agarose gel (2% wt./vol.) electrophoresis of the PCR products. Strains that tested negative for any of the Alp genes under investigation were considered non-typeable (NT).

### Pilus typing

To identify the presence of pilus islands in the strains, three PCR assays were used to detect the PI-1, PI-2a or PI-2b genes as described previously [20]. In brief, the PCR system (total volume, 25 µl) contained 10 ng DNA template, 1× PCR buffer; 2 mmol/l MgCl_2_, 200 µmol/l dNTPs, 400 nmol/l of each of the six primers; and 0.25 U of TaqDNA polymerase (Tiangen, China). The amplification conditions were as follows: preheating at 94°C for 5 min, followed by 35 cycles of 94°C for 45 s, 54°C for 45 s, and 72°C for 1 min and 30 s to 2 min (according to the lengths of the amplicons) and concluding with a cycle of 72°C for 10 min. The identity of the PCR products was analyzed by agarose gel (1% wt./vol.) electrophoresis and deemed to be positive based on the expected size of the PCR fragment.

### MLST

All the isolates were typed using multilocus sequence typing (MLST) in this study. The seven housekeeping genes (*adh*, *pheS*, *atr*, *glnA*, *sdhA*, *glcK* and *tkt*) were amplified by PCR and internal fragments sequences were obtained as described previously [7]. For each isolate, the allele number and sequence types (STs) were defined by analysis of the alleles sequence in the MLST database (http://pubmlst.org/sagalactiae/). The allele sequences or previously undescribed ST were assigned new numbers and the data were deposited in the MLST database. CC analysis was performed using the entire *S. agalactiae* MLST database and eBURST program (http://eburst.mlst.net) [30].

### PFGE

DNA was extracted and digested with the *Sma*I restriction enzyme (Takara, China.) as previously described [31,32]. The PFGE program was performed according to Chen et al. [32]. A *Salmonella* serotype Braenderup H9812 DNA digested with *Xba*I was used as the molecular size standard as recommended in PulseNet [33]. The *S. agalactiae* PFGE patterns were analyzed with BioNumerics (Applied Maths BVBA, Belgium) using an optimization setting of 0.00% and band position tolerance of 1.5%. Cluster analysis was performed using the Dice coefﬁcient and UPGMA of the digitalized PFGE patterns for the 102 *S. agalactiae* strains. Genotypic related groups, characterized at 80% similarity or above are represented by dashed rectangles in the dendrogram.

### Statistical analysis

Categorical data were analyzed by using the Pearson chi-squared test. When data were insuﬃcient for the test demands, Fisher’s exact test and Likelihood-ratio tests were used. P values of <0.05 were considered to indicate statistical significance.

## Supporting Information

Table S1 Characteristics of *Streptococcus agalactiae* isolates from bovine subclinical mastitis in Eastern China.
(DOC)

Table S2 Information of the 33 commercial dairy farms investigated in this study.
(DOC)
