# Supplementary material for: Molecular Characterization of Streptococcus agalactiae Isolated from Bovine Mastitis in Eastern China
Source: PLoS One. 2013 Jul 10;8(7):e67755. doi: 10.1371/journal.pone.0067755 (PMC3707890; doi:10.1371/journal.pone.0067755)
Supplement: Table S1 — Characteristics of Streptococcus agalactiae isolates from bovine subclinical mastitis in Eastern China. (DOC) [file pone.0067755.s001.doc]

**Supplementary Table S1.**

Characteristics of *Streptococcus agalactiae* isolates from bovine subclinical mastitis in eastern China

| Farm code | Province or municipality | Isolate code | Allelic proﬁles | | | | | | | ST^a^ | CCs^b^ | Capsular genotype | Alp^c^ | PI^d^ |
| --- | --- | --- | --- | --- | --- | --- | --- | --- | --- | --- | --- | --- | --- | --- |
|  |  |  | *adhP* | *pheS* | *atr* | *glnA* | *sdhA* | *glcK* | *tkt* |  |  |  |  |  |
| NJ-G | Jiangsu | ZJ201101 | 16 | 1 | 6 | 2 | 51^e^ | 9 | 2 | 568^f^ | 103 | Ⅰa | NT^g^ | 2b |
| NJ-G | Jiangsu | ZJ201102 | 16 | 1 | 6 | 2 | 51^e^ | 9 | 2 | 568^f^ | 103 | Ⅰa | NT^g^ | 2b |
| NJ-G | Jiangsu | ZJ201103 | 16 | 1 | 6 | 2 | 51^e^ | 9 | 2 | 568^f^ | 103 | Ⅰa | NT^g^ | 2b |
| NJ-H | Jiangsu | JSCH201102 | 16 | 1 | 6 | 2 | 51^e^ | 9 | 2 | 568^f^ | 103 | Ⅰa | NT^g^ | 2b |
| NJ-G | Jiangsu | ZJ201104 | 16 | 1 | 6 | 2 | 51^e^ | 9 | 2 | 568^f^ | 103 | Ⅰa | NT^g^ | 2b |
| NJ-G | Jiangsu | ZJ201105 | 16 | 1 | 6 | 2 | 51^e^ | 9 | 2 | 568^f^ | 103 | Ⅰa | NT^g^ | 2b |
| NJ-G | Jiangsu | ZJ201106 | 16 | 1 | 6 | 2 | 51^e^ | 9 | 2 | 568^f^ | 103 | Ⅰa | NT^g^ | 2b |
| NJ-G | Jiangsu | ZJ201107 | 16 | 1 | 6 | 2 | 51^e^ | 9 | 2 | 568^f^ | 103 | Ⅰa | NT^g^ | 2b |
| NJ-G | Jiangsu | ZJ201108 | 16 | 1 | 6 | 2 | 51^e^ | 9 | 2 | 568^f^ | 103 | Ⅰa | NT^g^ | 2b |
| NJ-G | Jiangsu | ZJ201109 | 16 | 1 | 6 | 2 | 51^e^ | 9 | 2 | 568^f^ | 103 | Ⅰa | NT^g^ | 2b |
| NJ-B | Jiangsu | TY201102 | 16 | 1 | 6 | 2 | 51^e^ | 9 | 2 | 568^f^ | 103 | Ⅰa | NT^g^ | 2b |
| NJ-C | Jiangsu | XG201103 | 16 | 1 | 6 | 2 | 51^e^ | 9 | 2 | 568^f^ | 103 | Ⅰa | NT^g^ | 2b |
| NJ-C | Jiangsu | XG201104 | 16 | 1 | 6 | 2 | 51^e^ | 9 | 2 | 568^f^ | 103 | Ⅰa | NT^g^ | 2b |
| NJ-E | Jiangsu | TQ2011050512 | 16 | 1 | 6 | 2 | 51^e^ | 9 | 2 | 568^f^ | 103 | Ⅰa | NT^g^ | 2b |
| NJ-E | Jiangsu | TQ2011050513 | 16 | 1 | 6 | 2 | 51^e^ | 9 | 2 | 568^f^ | 103 | Ⅰa | NT^g^ | 2b |
| NJ-E | Jiangsu | TQ2011050514 | 16 | 1 | 6 | 2 | 51^e^ | 9 | 2 | 568^f^ | 103 | Ⅰa | NT^g^ | 2b |
| NJ-E | Jiangsu | TQ2011050515 | 16 | 1 | 6 | 2 | 51^e^ | 9 | 2 | 568^f^ | 103 | Ⅰa | NT^g^ | 2b |
| NJ-D | Jiangsu | NJZJ2011050501 | 16 | 1 | 1 | 2 | 1 | 1 | 5 | 570^f^ | 64 | Ⅱ | Alp4 | 2b |
| NJ-D | Jiangsu | NJZJ2011050502 | 16 | 1 | 1 | 2 | 1 | 1 | 5 | 570^f^ | 64 | Ⅱ | Alp4 | 2b |
| TZ-F | Jiangsu | TZ201101 | 16 | 1 | 6 | 2 | 51^e^ | 9 | 2 | 568^f^ | 103 | Ⅰa | NT^g^ | 2b |
| TZ-F | Jiangsu | TZ201102 | 16 | 1 | 6 | 2 | 51^e^ | 9 | 2 | 568^f^ | 103 | Ⅰa | NT^g^ | 2b |
| TZ-F | Jiangsu | TZ201103 | 16 | 1 | 6 | 2 | 51^e^ | 9 | 2 | 568^f^ | 103 | Ⅰa | NT^g^ | 2b |
| TZ-F | Jiangsu | TZ201104 | 16 | 1 | 6 | 2 | 51^e^ | 9 | 2 | 568^f^ | 103 | Ⅰa | NT^g^ | 2b |
| TZ-F | Jiangsu | TZ201107 | 16 | 1 | 6 | 2 | 51^e^ | 9 | 2 | 568^f^ | 103 | Ⅰa | NT^g^ | 2b |
| TZ-F | Jiangsu | TZ201108 | 16 | 1 | 6 | 2 | 51^e^ | 9 | 2 | 568^f^ | 103 | Ⅰa | NT^g^ | 2b |
| NJ-A | Jiangsu | CH201104 | 16 | 1 | 6 | 2 | 51^e^ | 9 | 2 | 568^f^ | 103 | Ⅰa | NT^g^ | 2b |
| XZ-J | Jiangsu | XY201102 | 16 | 1 | 6 | 2 | 51^e^ | 9 | 2 | 568^f^ | 103 | Ⅰa | NT^g^ | 2b |
| XZ-J | Jiangsu | XY201103 | 13 | 1 | 1 | 13 | 1 | 28 | 5 | 301 | 67 | Ⅱ | Alp1 | 2b |
| XZ-J | Jiangsu | XY201104 | 13 | 1 | 1 | 13 | 1 | 28 | 5 | 301 | 67 | Ⅱ | Alp1 | 2b |
| XZ-J | Jiangsu | XY201105 | 13 | 1 | 1 | 13 | 1 | 28 | 5 | 301 | 67 | Ⅱ | Alp1 | 2b |
| XZ-J | Jiangsu | XY201107 | 13 | 1 | 1 | 2 | 1 | 28 | 5 | 313 | 67 | Ⅱ | Alp1 | 2b |
| HA-K | Jiangsu | HAJL2011070601 | 16 | 1 | 6 | 2 | 51^e^ | 9 | 2 | 568^f^ | 103 | Ⅰa | NT^g^ | 2b |
| HA-K | Jiangsu | HAJL2011070602 | 13 | 1 | 1 | 13 | 1 | 28 | 5 | 301 | 67 | Ⅱ | Alp1 | 2b |
| LY-I | Jiangsu | LYG201102 | 16 | 1 | 6 | 2 | 9 | 9 | 2 | 103 | 103 | Ⅰa | NT^g^ | 2b |
| FY-N | Anhui | BH201101 | 16 | 1 | 6 | 2 | 9 | 9 | 2 | 103 | 103 | Ⅰa | NT^g^ | 2b |
| FY-N | Anhui | BH201102 | 16 | 1 | 6 | 2 | 9 | 9 | 2 | 103 | 103 | Ⅰa | NT^g^ | 2b |
| FY-N | Anhui | BH201103 | 16 | 1 | 6 | 2 | 9 | 9 | 2 | 103 | 103 | Ⅰa | NT^g^ | 2b |
| FY-N | Anhui | BH201104 | 16 | 1 | 6 | 2 | 9 | 9 | 2 | 103 | 103 | Ⅰa | NT^g^ | 2b |
| FY-N | Anhui | BH201105 | 16 | 1 | 6 | 2 | 9 | 9 | 2 | 103 | 103 | Ⅰa | NT^g^ | 2b |
| FY-N | Anhui | BH201106 | 16 | 1 | 6 | 2 | 9 | 9 | 2 | 103 | 103 | Ⅰa | NT^g^ | 2b |
| FY-N | Anhui | BH201107 | 16 | 1 | 6 | 2 | 9 | 9 | 2 | 103 | 103 | Ⅰa | NT^g^ | 2b |
| FY-N | Anhui | BH201109 | 16 | 1 | 6 | 2 | 9 | 9 | 2 | 103 | 103 | Ⅰa | NT^g^ | 2b |
| FY-N | Anhui | BH201110 | 16 | 1 | 6 | 2 | 9 | 9 | 2 | 103 | 103 | Ⅰa | NT^g^ | 2b |
| FY-N | Anhui | BH201111 | 16 | 1 | 6 | 2 | 9 | 9 | 2 | 103 | 103 | Ⅰa | NT^g^ | 2b |
| FY-N | Anhui | BH201112 | 16 | 1 | 6 | 2 | 9 | 9 | 2 | 103 | 103 | Ⅰa | NT^g^ | 2b |
| FY-N | Anhui | BH201113 | 16 | 1 | 6 | 2 | 9 | 9 | 2 | 103 | 103 | Ⅰa | NT^g^ | 2b |
| FY-N | Anhui | BH201114 | 16 | 1 | 6 | 2 | 9 | 9 | 2 | 103 | 103 | Ⅰa | NT^g^ | 2b |
| FY-N | Anhui | BH201116 | 16 | 1 | 6 | 2 | 9 | 9 | 2 | 103 | 103 | Ⅰa | NT^g^ | 2b |
| FY-N | Anhui | BH201117 | 16 | 1 | 6 | 2 | 9 | 9 | 2 | 103 | 103 | Ⅰa | NT^g^ | 2b |
| FY-N | Anhui | BH201118 | 16 | 1 | 6 | 2 | 9 | 9 | 2 | 103 | 103 | Ⅰa | NT^g^ | 2b |
| FY-O | Anhui | BA201101 | 16 | 1 | 6 | 2 | 9 | 9 | 2 | 103 | 103 | Ⅰa | NT^g^ | 2b |
| FY-O | Anhui | BA201102 | 16 | 1 | 6 | 2 | 9 | 9 | 2 | 103 | 103 | Ⅰa | NT^g^ | 2b |
| FY-O | Anhui | BA201103 | 16 | 1 | 6 | 2 | 9 | 9 | 2 | 103 | 103 | Ⅰa | NT^g^ | 2b |
| FY-O | Anhui | BA201104 | 16 | 1 | 6 | 2 | 9 | 9 | 2 | 103 | 103 | Ⅰa | NT^g^ | 2b |
| FY-O | Anhui | BA201105 | 16 | 1 | 6 | 2 | 9 | 9 | 2 | 103 | 103 | Ⅰa | NT^g^ | 2b |
| FY-O | Anhui | BA201106 | 16 | 1 | 6 | 2 | 9 | 9 | 2 | 103 | 103 | Ⅰa | NT^g^ | 2b |
| FY-O | Anhui | BA201107 | 16 | 1 | 6 | 2 | 9 | 9 | 2 | 103 | 103 | Ⅰa | NT^g^ | 2b |
| FY-O | Anhui | BA201108 | 16 | 1 | 6 | 2 | 9 | 9 | 2 | 103 | 103 | Ⅰa | NT^g^ | 2b |
| FY-O | Anhui | BA201109 | 16 | 1 | 6 | 2 | 9 | 9 | 2 | 103 | 103 | Ⅰa | NT^g^ | 2b |
| FY-O | Anhui | BA201110 | 13 | 1 | 1 | 13 | 1 | 1 | 5 | 67 | 67 | Ⅱ | NT^g^ | 2b |
| FY-O | Anhui | BA201111 | 16 | 1 | 6 | 2 | 9 | 9 | 2 | 103 | 103 | Ⅰa | NT^g^ | 2b |
| FY-O | Anhui | BA201112 | 16 | 1 | 6 | 2 | 9 | 9 | 2 | 103 | 103 | Ⅰa | NT^g^ | 2b |
| FY-O | Anhui | BA201113 | 16 | 1 | 6 | 2 | 9 | 9 | 2 | 103 | 103 | Ⅰa | NT^g^ | 2b |
| FY-O | Anhui | BA201114 | 16 | 1 | 6 | 2 | 9 | 9 | 2 | 103 | 103 | Ⅰa | NT^g^ | 2b |
| WF-M | Shandong | WF201102 | 16 | 1 | 6 | 2 | 51^e^ | 9 | 2 | 568^f^ | 103 | Ⅰa | NT^g^ | 2b |
| HZ-P | Zhejiang | XC201101 | 16 | 1 | 6 | 2 | 9 | 9 | 2 | 103 | 103 | Ⅰa | NT^g^ | 2b |
| HZ-P | Zhejiang | XC201105 | 16 | 1 | 6 | 2 | 9 | 9 | 2 | 103 | 103 | Ⅰa | NT^g^ | 2b |
| HZ-P | Zhejiang | XC201106 | 16 | 1 | 1 | 2 | 9 | 9 | 2 | 103 | 103 | Ⅰa | NT^g^ | 2b |
| HZ-P | Zhejiang | XC201107 | 13 | 1 | 1 | 13 | 1 | 1 | 5 | 67 | 67 | Ⅰa | NT^g^ | 2b |
| HZ-P | Zhejiang | XC201108 | 16 | 1 | 6 | 2 | 9 | 9 | 2 | 103 | 103 | Ⅰa | NT^g^ | 2b |
| HZ-P | Zhejiang | XC201109 | 13 | 1 | 1 | 13 | 1 | 1 | 5 | 67 | 67 | Ⅱ | NT^g^ | 2b |
| HZ-P | Zhejiang | XC201110 | 13 | 1 | 1 | 13 | 1 | 1 | 5 | 67 | 67 | Ⅱ | NT^g^ | 2b |
| HZ-P | Zhejiang | XC201112 | 13 | 1 | 1 | 13 | 1 | 1 | 5 | 67 | 67 | Ⅱ | NT^g^ | 2b |
| HZ-P | Zhejiang | XC201113 | 16 | 1 | 6 | 2 | 9 | 9 | 2 | 103 | 103 | Ⅰa | NT^g^ | 2b |
| HZ-R | Zhejiang | HJ201101 | 16 | 1 | 6 | 2 | 9 | 9 | 2 | 103 | 103 | Ⅰa | NT^g^ | 2b |
| HZ-R | Zhejiang | HJ201102 | 16 | 1 | 6 | 2 | 9 | 9 | 2 | 103 | 103 | Ⅰa | NT^g^ | 2b |
| HZ-R | Zhejiang | HJ201103 | 16 | 1 | 6 | 2 | 9 | 9 | 2 | 103 | 103 | Ⅰa | NT^g^ | 2b |
| NC-Q | Jiangxi | NC201101 | 16 | 1 | 6 | 2 | 9 | 9 | 2 | 103 | 103 | Ⅰa | NT^g^ | 2b |
| NC-Q | Jiangxi | NC201102 | 16 | 1 | 6 | 2 | 9 | 9 | 2 | 103 | 103 | Ⅰa | NT^g^ | 2b |
| NC-Q | Jiangxi | NC201103 | 16 | 1 | 6 | 2 | 9 | 9 | 2 | 103 | 103 | Ⅰa | NT^g^ | 2b |
| NJ-L | Jiangsu | XGT201101 | 16 | 1 | 6 | 2 | 51^e^ | 9 | 2 | 568^f^ | 103 | Ⅰa | NT^g^ | 2b |
| NJ-S | Jiangsu | TYT201101 | 16 | 1 | 6 | 2 | 51^e^ | 9 | 2 | 568^f^ | 103 | Ⅰa | NT^g^ | 2b |
| NJ-S | Jiangsu | TYT201102 | 16 | 1 | 6 | 2 | 51^e^ | 9 | 2 | 568^f^ | 103 | Ⅰa | NT^g^ | 2b |
| NJ-S | Jiangsu | TYT201103 | 16 | 1 | 6 | 2 | 51^e^ | 9 | 2 | 568^f^ | 103 | Ⅰa | NT^g^ | 2b |
| NJ-S | Jiangsu | TYT201104 | 16 | 1 | 6 | 2 | 51^e^ | 9 | 2 | 568^f^ | 103 | Ⅰa | NT^g^ | 2b |
| SH-AF | Shanghai | SH201101 | 16 | 1 | 6 | 2 | 9 | 9 | 2 | 103 | 103 | Ⅰa | NT^g^ | 2b |
| SH-AF | Shanghai | SH201102 | 16 | 1 | 6 | 2 | 9 | 9 | 2 | 103 | 103 | Ⅰa | NT^g^ | 2b |
| SH-AF | Shanghai | SH201103 | 16 | 1 | 6 | 2 | 9 | 9 | 2 | 103 | 103 | Ⅰa | NT^g^ | 2b |
| SH-AF | Shanghai | SH201104 | 16 | 1 | 6 | 2 | 9 | 9 | 2 | 103 | 103 | Ⅰa | NT^g^ | 2b |
| SH-AF | Shanghai | SH201105 | 16 | 1 | 6 | 2 | 9 | 9 | 2 | 103 | 103 | Ⅰa | NT^g^ | 2b |
| SH-AF | Shanghai | SH201106 | 16 | 1 | 6 | 2 | 9 | 9 | 2 | 103 | 103 | Ⅰa | NT^g^ | 2b |
| SH-AF | Shanghai | SH201107 | 16 | 1 | 6 | 2 | 9 | 9 | 2 | 103 | 103 | Ⅰa | NT^g^ | 2b |
| SH-AF | Shanghai | SH201108 | 16 | 1 | 6 | 2 | 9 | 9 | 2 | 103 | 103 | Ⅰa | NT^g^ | 2b |
| SH-AF | Shanghai | SH201109 | 16 | 1 | 6 | 2 | 9 | 9 | 2 | 103 | 103 | Ⅰa | NT^g^ | 2b |
| SH-AF | Shanghai | SH2011010 | 16 | 1 | 6 | 2 | 9 | 9 | 2 | 103 | 103 | Ⅰa | NT^g^ | 2b |
| SH-AG | Shanghai | SH2011015 | 16 | 1 | 6 | 2 | 9 | 9 | 2 | 103 | 103 | Ⅰa | NT^g^ | 2b |
| SH-AG | Shanghai | SH2011016 | 16 | 1 | 6 | 2 | 9 | 9 | 2 | 103 | 103 | Ⅰa | NT^g^ | 2b |
| SH-AG | Shanghai | SH2011017 | 16 | 1 | 6 | 2 | 9 | 9 | 2 | 103 | 103 | Ⅰa | NT^g^ | 2b |
| SH-AG | Shanghai | SH2011018 | 16 | 1 | 6 | 2 | 9 | 9 | 2 | 103 | 103 | Ⅰa | NT^g^ | 2b |
| SH-AG | Shanghai | SH2011019 | 16 | 1 | 6 | 2 | 9 | 9 | 2 | 103 | 103 | Ⅰa | NT^g^ | 2b |
| SH-AG | Shanghai | SH2011020 | 16 | 1 | 6 | 2 | 9 | 9 | 2 | 103 | 103 | Ⅰa | NT^g^ | 2b |
| SH-AG | Shanghai | SH2011021 | 16 | 1 | 6 | 2 | 9 | 9 | 2 | 103 | 103 | Ⅰa | NT^g^ | 2b |
| ^a^ST, sequence type. ^b^CC, clonal complex. ^c^Alp, alpha-like protein. ^d^PI, pilus island. ^e^Novel allelic profiles. ^f^Novel sequence types (ST). ^g^NT, none-typeable | | | | | | | | | | | | | | |
