# Supplementary material for: Molecular Characterization of Streptococcus agalactiae Isolated from Bovine Mastitis in Eastern China
Source: PLoS One. 2013 Jul 10;8(7):e67755. doi: 10.1371/journal.pone.0067755 (PMC3707890; doi:10.1371/journal.pone.0067755)
Supplement: Table S2 — Information of the 33 commercial dairy farms investigated in this study. (DOC) [file pone.0067755.s002.doc]

| **Cities** | **Province or municipality** | **Farm code** | **Number of lactating cows** | **total milk yield per year(kg)** | ***s. agalactiae* positive cows** | | |
| --- | --- | --- | --- | --- | --- | --- | --- |
|  |  |  |  |  | **Number of examined cows** | **Number of positives** | **Frequency （%）** |
| NanJing | Jiangsu | NJ-A | 201 | 1632924 | 9 | 1 | (11) |
|  | Jiangsu | NJ-B | 200 | 1616500 | 17 | 1 | (6) |
|  | Jiangsu | NJ-C | 226 | 1796926 | 23 | 2 | (9) |
|  | Jiangsu | NJ-D | 298 | 2291322 | 15 | 2 | (13 |
|  | Jiangsu | NJ-E | 409 | 3122832 | 11 | 4 | (36) |
|  | Jiangsu | NJ-G | 132 | 1006236 | 20 | 9 | (45) |
|  | Jiangsu | NJ-H | 201 | 1534032 | 15 | 1 | (7) |
|  | Jiangsu | NJ-L | 200 | 1573000 | 19 | 1 | (5) |
|  | Jiangsu | NJ-S | 395 | 3019775 | 13 | 4 | (31) |
| TaiZhou | Jiangsu | TZ-F | 503 | 3949556 | 21 | 6 | (29) |
| LianYungang | Jiangsu | LY-I | 109 | 838101 | 12 | 1 | (8) |
| XuZhou | Jiangsu | XZ-J | 350 | 2625350 | 26 | 5 | (19) |
| HuaiAn | Jiangsu | HA-K | 252 | 1979208 | 29 | 2 | (7) |
| WeiFang | Shandong | WF-M | 99 | 762100 | 12 | 1 | (8) |
|  | Shandong | WF-U | 212 | 1665472 | 11 | 0 | (0) |
|  | Shandong | WF-V | 135 | 1060290 | 17 | 0 | (0) |
|  | Shandong | WF-W | 185 | 1399340 | 15 | 0 | 0 |
|  | Shandong | WF-AC | 176 | 1382392 | 12 | 0 | (0) |
| FengYang | Anhui | FY-N | 351 | 2619162 | 28 | 16 | (57) |
|  | Anhui | FY-O | 300 | 2179500 | 24 | 14 | (58) |
| HangZhou | Zhejiang | HZ-P | 200 | 1484270 | 30 | 9 | (30) |
|  | Zhejiang | HZ-AB | 223 | 1712327 | 28 | 0 | (0) |
|  | Zhejiang | HZ-R | 345 | 2724199 | 11 | 3 | (27) |
|  | Zhejiang | HZ-AA | 214 | 1646580 | 11 | 0 | (0) |
| ShuYang | Jiangsu | SY-T | 215 | 1689040 | 29 | 0 | (0) |
|  | Jiangsu | ZZ-X | 420 | 3169320 | 24 | 0 | (0) |
| NanPing | Fujian | NP-Y | 255 | 1928565 | 15 | 0 | (0) |
|  | Fujian | NP-Z | 230 | 1770588 | 17 | 0 | (0) |
| NanChang | Jiangxi | NC-Q | 212 | 1610776 | 37 | 3 | (8) |
| ZiBo | Shandong | ZB-AE | 132 | 996415 | 11 | 0 | (0) |
|  | Shandong | ZB-AD | 152 | 1162784 | 14 | 0 | (0) |
| Shanghai | Shanghai | SH-AF | 112 | 843809 | 21 | 10 | (48) |
|  | Shanghai | SH-AG | 201 | 1520163 | 22 | 7 | (32) |
|  |  | Total | 7845 | 60312854 | 619 | 102 | (16) |

**Supplementary Table S2.**

Information of the 33 commercial dairy farms studied in this study.
